# Viral wheezing in early childhood as a risk factor for asthma in young adulthood: A prospective long‐term cohort study

**DOI:** 10.1002/hsr2.538

**Published:** 2022-03-07

**Authors:** Paula Heikkilä, Matti Korppi, Marja Ruotsalainen, Katri Backman

**Affiliations:** ^1^ Department of Pediatrics, Tampere Center for Child Health Research, Faculty of Medicine and Health Technology Tampere University and Tampere University Hospital Tampere Finland; ^2^ Institute of Clinical Medicine University of Eastern Finland Kuopio Finland; ^3^ Department of Pediatrics Kuopio University Hospital and University of Eastern Finland Kuopio Finland

**Keywords:** asthma, cohort study, risk factors, viral wheezing

## Abstract

**Background and Aims:**

There is an increased risk of asthma after viral wheezing episodes in early childhood, but unfortunately, prospective longitudinal data until adulthood are mostly lacking. The aim of this cohort study was to evaluate the risk of asthma in young adulthood after hospitalization for viral wheezing episodes in early childhood.

**Methods:**

The original cohort comprised 100 individuals aged <24 months who were hospitalized for viral wheezing episodes in 1992–1993. After the index episode, data on a diagnosis of asthma 1 year later and at median ages of 4.0, 7.2, and 12.3 years were recorded in follow‐up visits. Forty‐nine individuals attended the latest follow‐up visit at the age of 17–20 years. Current asthma was diagnosed based on symptoms at the time of the last follow‐up, use of inhaled corticosteroids and peak expiratory flow monitoring.

**Results:**

A total of 26 (53%) of the 49 cohort individuals had asthma at a mean age of 18.8 years. In multivariate analyses, a diagnosis of asthma 1 year after index hospitalization and at ages 4.0, 7.2, and 12.3 years were significant risk factors for current asthma (adjusted odds ratios [aORs] of 7.13, 8.86, 8.05, and 21.16, respectively). Atopic dermatitis in infancy (aOR: 4.20) and eosinophilia on admission (5.18) were also significant predictive factors for asthma.

**Conclusion:**

Over half (26/49) of the participants who had been hospitalized for viral wheezing episodes in early childhood had asthma in young adulthood. An asthma diagnosis at any age during childhood, as well as eosinophilia in early childhood, were independent significant predictive factors for asthma.

## 1 INTRODUCTION

Wheezing related to lower respiratory tract infections (LRTI) is common in infancy, with approximately one in every three children experiencing at least one wheezing episode during the first 3 years of life.^1^, ^2^ The first wheezing episode in infants aged under 24 months has traditionally been called bronchiolitis, although the age limit of bronchiolitis in most European countries is 12 months.^3^ Respiratory syncytial virus (RSV) is the most common cause of bronchiolitis in wheezing infants younger than 12 months, whereas rhinovirus is common in those wheezing child aged 12–24 months.^3^

Previous birth cohort and postbronchiolitis studies reported that bronchiolitis and wheezing in early childhood may have long‐term effects on respiratory health.^4^, ^5^ Wheezing and asthma symptoms, although common after bronchiolitis at preschool age, are usually getting infrequent at school age. However, after puberty, the symptoms may recur, even in those without any symptom recurrence after bronchiolitis.^4^ Long‐term follow‐up studies demonstrated an increased risk of asthma and impaired lung function continuing until adulthood after viral wheezing in early childhood.^6^, ^7^, ^8^, ^9^

The factors that influence the development of asthma are complex and remain, despite active research, poorly understood.^10^ A familial history of asthma, especially in the mother, exposure to tobacco smoke in infancy and the presence of atopic dermatitis in early life were found to be common risk factors for asthma after wheezing in the first 2 years of life in both birth cohort and postbronchiolitis studies.^4^, ^11^ In young children, specific laboratory markers, such as eosinophilia and a high immunoglobulin E (IgE) level, as well as early‐life sensitization to airborne allergens confirmed by skin prick tests (SPTs) or by measuring specific IgE to airborne allergens in serum samples, have been documented as predictors of asthma in later life in hospital‐based follow‐up studies.^12^

At preschool age, an asthma diagnosis is usually based on typical symptoms and asthma‐predictive risk factors.^13^ Therefore, a diagnosis of asthma in early childhood may be more uncertain than in later years (i.e., school age) when airflow obstruction can be confirmed by lung function tests.^13^

We followed up a cohort of 100 children aged less than 24 months at the time of hospitalization for viral wheezing episodes in Kuopio University Children's Hospital, Finland, in 1992–1993^14^ to early adulthood. The aim of this study was to evaluate early‐childhood and current risk factors for asthma in later life (i.e., aged 17–20 years) in this prospectively followed cohort. An additional aim was to describe the evolution on asthma from infancy through to early adulthood after hospitalization for viral wheezing in early‐childhood.

## 2 MATERIALS AND METHODS

### 2.1 Design of the cohort study

The original cohort consisted of 100 children aged younger than 24 months who were hospitalized in 1992–1993 because of a first wheezing episode associated with LRTI.^14^ The study was performed in Kuopio University Children's Hospital, Finland. After the index episode, the cohort was prospectively followed‐up at four control visits: 1 year after the index episode^14^ and again at median ages of 4.0 years,^15^ 7.2 years,^12^ and 12.3 years.^16^ In 2010, 49 cohort subjects aged 17–20 years attended the last follow‐up visit.^17^ Among them, RSV was detected in 14 cases and rhinovirus likewise in 14 cases during the primary hospitalization for wheezing in early childhood. Electronic Supporting Information Material 1 provides information on the asthma status of the participants from 1992 to 2010 (Figure S1). All the analyses in the current study included the data on the 49 participants who attended the clinical follow‐up visit in 2010 at a mean age of 18.8 years.

### 2.2 Data collected in early childhood

At the time of the index hospitalization episode, a venous blood sample was obtained for analysis of blood eosinophils, eosinophil cationic protein (ECP) in serum, supplemented with ECP measurement in nasopharyngeal aspirates, and total and allergen‐specific IgE in serum.^14^ Eosinophil counts ≥0.45 cells × 10^9^/L,^18^ serum ECP concentration ≥16 µg/L,^18^ nasopharyngeal ECP concentration ≥870 ng/g mucus^19^ and total serum IgE ≥ 60 kU/L^14^ were considered elevated. The detection limit in the measurement of allergen‐specific IgE was 0.35 µg/L.^20^ Respiratory viruses, including RSV and rhinovirus, were identified using antigen and genome‐detection methods. The associations between these viruses and outcomes have been published previously.^16^, ^21^

The parents of the recruited bronchiolitis patients were interviewed using structured questionnaires during hospitalization and at subsequent follow‐up visits 4 weeks and 4 months later.^18^ The questionnaires collected data on family histories of asthma and atopy, maternal and paternal histories of smoking, including maternal smoking during pregnancy, childhood histories of wheezing episodes and atopic dermatitis, and the presence of household pets. For both children and parents, only asthma and allergies diagnosed by a physician were registered.

### 2.3 Data collected in preschool and school age

At the follow‐up visit 1 year after the index episode requiring hospitalization, asthma was diagnosed if at least three episodes of physician‐confirmed wheezing during the preceding year were reported.^14^ In 1995, at a median age of 4.0 years, asthma was defined as the presence of a previous asthma diagnosis and at least one reported physician‐diagnosed wheezing episode during the preceding year or reported use of maintenance medication for asthma.^15^ In 1999 and 2004, at median ages of 7.2 and 12.3 years, respectively, asthma was diagnosed if continuous maintenance medication for asthma, or repeated wheezing episodes and/or a prolonged cough apart from the infection during the preceding 12 months were reported, and the result of an exercise challenge test was positive.^12^, ^16^

### 2.4 Current data collected in early adulthood

At the follow‐up visit in 2010, when the participants were aged 17–20 years, they completed a questionnaire, which included questions about asthma and allergy symptoms, asthma and allergy diagnoses, medication and current smoking.^17^ In addition, all the participants performed daily peak expiratory flow (PEF) measurements at home for 2 weeks before the study visit. The criteria for abnormal PEF were daily variability in PEF ≥ 20% or a bronchodilator response ≥15% at least twice during this 2 weeks period.^22^ A physician interviewed and examined all the participants and checked the questionnaire and confirmed the responses with the participants.^17^

Current asthma was defined as the presence of a previous asthma diagnosis, together with symptoms suggestive of asthma during the last 12 months. These symptoms included recurrent wheezing, a prolonged cough, a chronic night cough or continuous use of inhaled corticosteroids.^17^ In addition, participants who presented with abnormal home PEF monitoring results and reported asthma‐presumptive symptoms and/or repeated use of bronchodilators were considered to have current asthma.^17^ Current asthma was present in 26 (53%) of the 49 participants who attended the follow‐up visit in 2010 (Figure S1).

During the follow‐up visit, SPTs for the following allergens were performed: dog, cat, horse and cow dander, birch, common alder, Timothy grass and mugwort pollens, and three types of common dust mites.^17^ The presence of a positive SPT result for at least one allergen was defined as current atopic sensitization. Current allergy was defined as the presence of atopic sensitization and either atopic dermatitis, allergic conjunctivitis or allergic rhinitis.

### 2.5 Statistical analysis

The data were analyzed using IBM SPSS Statistics for Windows, version 25.0 software (IBM Corp.). Descriptive statistics are presented as numbers and proportions or medians, with 25%–75% interquartile ranges (IQRs). The Mann–Whitney *U*‐test was used for comparisons of continuous variables. Logistic regression was used for both univariate and multivariate analyses of categorical variables, and the results were presented as odds ratios (ORs), with their 95% confidence intervals (95% CIs). Multivariate models for the risk of current asthma were adjusted for current and early‐life confounding factors, when appropriate. The current confounders were sex and current daily smoking. The early confounders were age at the time of index hospital admission (<12 or ≥12 months), presence of household pets, or presence of pets in day care and passive smoke exposure during infancy. Two tailed tests were used, and *p* < 0.05 was regarded as statistically significant. The OR was statistically significant, if the lower limit of the 95% CI was over 1.00 (increased risk), or the upper limit, respectively, was under 1.0 (decreased risk).

### 2.6 Ethics

The study was approved by the Ethics committee of the Pohjois‐Savo Health Care District (Permission number: 76/2009), and written informed consent was obtained from all the participants or their parents.

## 3 RESULTS

Current atopic sensitization (OR: 5.04) and current allergy (OR: 6.25) were associated with an increased risk of current asthma in young adulthood (Table 1). Other potential current risk factors (i.e., sex and daily smoking), were not significantly associated with asthma (Table 1). Both current atopic sensitization (adjusted OR: 4.91, 95% CI: 1.25–19.36) and current allergy (6.08, 1.70–21.74) remained statistically significant risk factors for asthma in the multivariate analyses, adjusted for sex, and current tobacco smoking.

**Table 1:** Current risk factors for asthma among cohort subjects (*n* = 49) aged 17–20 years

Factors	Asthma, *n* = 26 (53%)	No asthma, *n* = 23 (47%)	Logistic regression^a^, *p* OR (95% CI)
Sex (male)	15 (57.7%)	15 (65.2%)	0.6
			1.38 (0.43–4.38)
Current atopic sensitization	22 (84.6%)	12 (52.2%)	**0.02**
			**5.04 (1.32–19.32)**
Current allergy	20 (76.9%)	8 (34.8%)	**0.003**
			**6.25 (1.79–21.87)**
Current daily smoking	7 (26.9%)	9 (39.1%)	0.4
			0.57 (0.17–1.91)

*Note*: For definitions of current allergy and atopic sensitization, see the text. Bold values indicates the statistical significance.

Abbreviations: OR, odds ratio; 95% CI, 95% confidence interval.

^a^ Logistic regression, no adjustments.

Asthma in parents (OR: 5.56) was a significant risk factor for current asthma, whereas asthma in mothers alone was not, although the OR was high (5.24) (Table 2). Atopic dermatitis in early childhood (4.07) was a significant asthma‐predictive factor. Other potential risk factors, including allergies in parents, age at the time of index hospital admission in early childhood, passive smoke exposure or the presence of household pets in infancy, and the rhinovirus or RSV etiology of early‐childhood wheezing did not show significant associations with current asthma at a mean age of 18.8 years (Table 2).

**Table 2:** Familial and early childhood risk factors in cohort study subjects (*n* = 49) for asthma at the age of 17–20 years

Factors	Current asthma, *n* = 26 (53%)	No asthma, *n* = 23 (47%)	Logistic regression, *p* OR (95% CI)
Maternal asthma	5 (19.3%)	1 (4.3%)	0.1
			5.24 (0.56–48.65)
Paternal asthma	5 (19.3%)	1 (4.3%)	0.2
			5.34 (0.56–48.65)
Parental asthma	9 (34.6%)	2 (8.7%)	**0.03**
			**5.56 (1.06–29.24)**
Maternal atopy	9 (34.6%)	9 (39.1%)	0.7
			0.824 (0.26–2.64)
Paternal atopy	7 (26.9%)	3 (13.0%)	0.3
			2.46 (0.55–10.91)
Parental atopy	14 (53.8%)	9 (39.1%)	0.3
			1.82 (0.58–5.67)
Early life atopic dermatitis	12 (46.2%)	4 (17.4%)	**0.03**
			**4.07 (1.08–15.33)**
Passive smoke exposure during infancy	15 (57.7%)	10 (43.5%)	0.3
			1.77 (0.57–5.51)
Maternal smoking during pregnancy	7 (26.9%)	4 (17.4%)	0.4
			1.75 (0.44–6.89)
Age at the time of index hospitalization (>12 months)	12 (46.2%)	9 (39.1%)	0.6
			1.33 (0.43–4.16)
Household pets or pets in day care in early childhood	7 (26.9%)	8 (34.8%)	0.6
			0.69 (0.20–2.34)
Rhinovirus	9 (34.6%)	5 (21.7%)	0.5
			1.80 (0.47–6.85)^a^
RSV	6 (23.1%)	8 (34.8%)	0.4
			0.47 (0.13–1.76)^b^

*Note*: Logistic regression, no adjustments; rhinovirus was detected in 14 and respiratory syncytial virus (RSV) in 14 cases. Bold values indicates the statistical significance.

Abbreviations: 95% CI, 95% confidence interval; OR, odds ratio.

^a^ Versus those 35 without rhinovirus.

^b^ Versus those 35 without RSV.

Neither the serum level of total IgE nor the serum level of specific IgE to inhalant or food allergens at the time of index hospital admission in early childhood were associated with asthma in young adulthood (Table 3). Elevated eosinophil counts (≥0.45 × 10^9^/L) at the time of index hospital admission in early childhood were significant predictor (OR: 4.52) of asthma in young adulthood (Table 3). ECP concentrations in serum or mucus samples taken at the time of index hospital admission were not associated with asthma risk in young adulthood (Table 3).

**Table 3:** Association of laboratory markers of atopy and eosinophilic activity at the time of index hospital admission with asthma aged 17–20 years

Parameters	Current asthma, *n* = 26 (53%)	No asthma, *n* = 23 (47%)	Statistical significance,^a^ *p* OR (95% CI)
Serum total IgE, kU/L, median (IQR)^b^	28.0 (3.5–87.75)	9.0 (1.0‐46.5)	0.2
Serum total IgE ≥60 kU/L, *n* (%)	7/24 (29.2%)	4/21 (19.0%)	0.4
			1.75 (0.43–7.10)
Specific IgE to inhaled allergens, *n* (%)	5/22 (22.7%)	3/20 (15.0%)	0.7
			1.67 (0.34–8.10)
Specific IgE to food allergens, *n* (%)	9/22 (40.9%)	7/20 (35.0%)	0.7
			1.29 (0.37–4.50)
Blood eosinophils × 10^9^/L, median (IQR)^c^	0.43 (0.12–0.70)	0.14 (0.07–0.34)	**0.02**
Eosinophils ≥ 0.45 × 10^9^/L, *n* (%)	10/24 (41.7%)	3/22 (13.6%)	**0.04**
			**5.52 (1.05–19.54)**
Serum ECP, µg/L, median (IQR)^d^	4.5 (2.2‐9.4)	4.5 (2.3–8.0)	0.8
Nasopharyngeal ECP, ng/g, median (IQR)^c^	347 (171–565)	498 (216–991)	0.1
Serum ECP ≥ 16 µg/L, *n*, (%)	4/25 (16.0%)	3/23 (13.0%)	0.8
			1.27 (0.252–6.40)
Nasopharyngeal ECP ≥ 870 ng/g, *n* (%)	4/23 (17.4%)	6/23 (26.1%)	0.5
			0.60 (01.14–2.48)

*Note*: Bold values indicates the statistical significance.

Abbreviations: 95% CI, 95% confidence interval; ECP, eosinophil cationic protein; IgE, immunoglobulin E; IQR, interquartile range (25%–75%); OR, odds ratio.

^a^ Mann–Whitney *U*‐test for continuous variables, logistic regression for categorical variables, no adjustments.

^b^ *n* = 45.

^c^ *n* = 46.

^d^ *n* = 46.

Wheezing episodes (OR: 5.14) and asthma diagnoses (OR: 5.54) at the 1‐year follow‐up after the index hospital admission and at subsequent follow‐ups at mean ages of 4.0 years (OR: 6.00), 7.2 years (OR: 6.22), and 12.3 years (OR: 8.00) increased the current asthma risk (Table 4).

**Table 4:** Association of wheezing and asthma after index hospitalization required viral wheezing episode in childhood with asthma in early adulthood

Factors	Current asthma, *n* = 26 (53%)	No asthma, *n* = 23 (47%)	Logistic regression, *p* OR (95% CI)
Wheezing 1 year after index hospitalization	18 (69.2%)	7 (30.4%)	**0.007**
			**5.14 (1.52–17.38)**
Asthma 1 year after index hospitalization	14 (53.8%)	4 (17.4%)	**0.008**
			**5.54 (1.47–20.86)**
Asthma at a median age of 4.0 years	18/24 (75.0%)	7/21 (33.3%)	**0.005**
			**6.00 (1.64–21.90)**
Asthma at a median age of 7.2 years	14/23 (60.9%)	4/20 (20.0%)	**0.007**
			**6.22 (1.57–24.71)**
Asthma at a median age of 12.3 years	15/25 (60.0%)	3/19 (15.8%)	**0.003**
			**8.00 (1.84–34.79)**
Asthma before or at a median age of 12.3 years	22 (84.6%)	7 (30.4%)	**<0.001**
			**11.79 (2.93–47.45)**

*Note*: For asthma definitions, see the text. Logistic regression, no adjustments. Bold values indicates the statistical significance.

Abbreviations: 95% CI, 95% confidence interval; OR, odds ratio.

Multivariate analyses were adjusted for sex, current daily smoking, and age at the time of index hospital admission, in addition to passive smoke exposure during infancy, and the presence of household pets or pets in day care during early childhood. As shown by the results, atopic dermatitis in infancy (adjusted OR: 4.20) and blood eosinophilia at the time of index hospital admission (adjusted OR: 5.18) remained statistically significant risk factors for asthma in young adulthood (Table 5). However, parental asthma marginally lost its statistical significance, although the adjusted OR was high (4.18). Asthma diagnoses at the follow‐up 1 year after index hospital admission (adjusted OR: 7.13) and at the follow‐ups when the participants were aged 4.0 years (adjusted OR: 8.86), 7.2 years (adjusted OR: 8.05) and 12.3 years (adjusted OR: 21.16) remained significant predictors of current asthma.

**Table 5:** Multivariate analyses of early‐life risk factors and previous asthma diagnoses in relation to adulthood asthma

Risk factors	Current asthma, OR (95% CI)
Parental asthma	4.18 (0.68–25.74)
Parental allergy	1.88 (0.52–6.74)
Atopic dermatitis in infancy	**4.20 (1.00–17.66)**
Blood eosinophils ≥ 0.45 × 10^9^/L	**5.18 (1.04–25.91)**
Asthma at the age of 1 year	**7.13 (1.58–32.24)**
Asthma at the age of 4.0 years	**8.86 (1.57–49.98)**
Asthma at the age of 7.2 years	**8.05 (1.64–39.66)**
Asthma at the age of 12.3 years	**21.16 (2.61–171.50)**
Asthma at least once before 17 years	**18.08 (3.23–101.08)**

*Note*: Logistic regression adjusted for sex, age at the time of index hospital admission, presence of household pets or pets in day care in infancy, exposure to tobacco smoke in infancy and daily smoking in young adulthood. Bold values indicates the statistical significance.

## 4 DISCUSSION

There were three primary findings of this prospective cohort study on predictive factors for asthma in young adults after hospitalization for viral wheezing episodes aged younger than 24 months. First, early life wheezing, an asthma diagnosis 1 year after the index hospital admission and physician‐diagnosed asthma at median ages of 4.0, 7.2, and 12.3 years were consistently associated with asthma at a mean age of 18.8 years (*n* = 49). Second, asthma in parents and atopic dermatitis in infancy were associated with asthma in early adulthood. Third, elevated blood eosinophils on the index hospital admission for viral wheezing episodes aged <24 months were associated with asthma in early adulthood. No such associations were found for other markers of eosinophilic activity or laboratory markers of atopy. As expected, asthma in early adulthood was associated with current allergy and atopic sensitization. When the analyses were repeated and adjusted for sex, age on admission, contact with household pets in infancy, early‐life exposure to tobacco smoke and current daily smoking, asthma in parents marginally lost its significance. As we reported previously,^17^ RSV and especially rhinovirus infections, linked to the index wheezing episode requiring hospitalization, were associated with asthma in early adulthood compared to population controls, but not in the present study with internal, within‐cohort controls.

In the present study, an asthma diagnosis at any of the four follow‐up visits until a mean age of 12.3 years was associated with an increased risk of asthma at a mean age of 18.8 years. In a Swedish postbronchiolitis cohort, the risk of asthma in later life (i.e., 27 years) was 10‐fold higher than in population‐based controls.^7^ In the same study, although an asthma diagnosis at the age of 5 or 10 years did not predict asthma in adulthood, an asthma diagnosis at the age of 18 years increased the risk of asthma at the age of 27 years significantly to 6.5‐fold.^7^ In the present cohort study, the risk of asthma aged 18.8 years was increased most (22‐fold) among those with asthma aged 12.3 years. This finding is in line with the current consensus on asthma evolution and age,^23^ that early‐childhood wheezing and asthma seem to recover at school age.^6^, ^7^ The latter does not imply total recovery but rather symptom remission, and relapses are common after puberty and in early adulthood.^6^, ^7^ Such relapses may remain undiagnosed because adolescents and young adults are reluctant to consult a doctor for asthma‐related symptoms due to denying their symptoms and negative perceptions of the disease among their peers.^4^

In previous birth cohort and postbronchiolitis studies, asthma in family members, especially mothers, was the most common risk factor for asthma in later life,^1^, ^4^, ^7^ as found in the present cohort. In a meta‐analysis, which included 18 studies from good‐quality systematic reviews, maternal asthma was associated with a 3.2‐fold risk and paternal asthma with a 2.6‐fold risk of physician‐diagnosed asthma aged 5–18 years.^11^ Prenatal environmental tobacco smoke exposures and premature births, particularly very preterm births, were other significant early‐life risk factors for later asthma.^11^ Although some studies found that atopic dermatitis in infancy was associated with asthma in later life, this association was not constant.^4^, ^7^

Tobacco smoke exposure during prenatal and early childhood periods are well‐known risk factors for bronchiolitis or viral wheezing in early life and asthma and lung function deficiency even in adulthood.^23^ However, neither maternal smoking nor passive smoke exposure in early childhood was associated with asthma in young adulthood in the present study. This finding may be explained by early exposure to tobacco smoke increasing the likelihood of hospitalization for viral wheezing episodes at young age and decreasing the statistical power for asthma prediction in later years in the within‐cohort analyses. Slightly more than half (51.0%) of the study group had exposure to tobacco smoke during infancy, and 48% of these individuals were current smokers at the time of last follow‐up visit. The prevalence of smoking in our study group far exceeded that of the current prevalence of smoking (mean: 11%) among young Finnish adults.^24^

In algorithms aimed at predicting the risk of asthma in school‐aged children, a high eosinophil count (i.e., an absolute count of ≥0.45 × 10^9^/L) at preschool age has been linked to an elevated risk of asthma at later life.^25^ It should be noted that eosinophil sampling during an infection, as in the present study, is not optimal, as infections often trigger a decrease in eosinophils.^26^ Thus, the finding of a normal eosinophil count in such cases would actually be an abnormal finding.^26^

The association of asthma with respiratory allergies is well known and is termed united airway disease.^27^ Previous Swedish and Finnish cohort studies investigated risk factors for asthma in 46 patients with a diagnosis of RSV‐related bronchiolitis in childhood who were followed up at a mean age of 18 years^8^ and 82 patients with a diagnosis of bronchiolitis in childhood who were followed up at a mean age of 27 years, resepectively.^9^ Both studies reported that current allergy, especially allergic rhinoconjunctivitis, was a significant risk factor for asthma in adulthood.^8^, ^9^ In the present cohort, more than 75% of those with current asthma reported allergic rhinitis and/or were sensitized to airborne allergens.

RSV and especially rhinovirus infections, when associated with wheezing in early childhood, are known to be associated with an increased risk of asthma in later life.^3^, ^11^, ^28^ On the other hand, a Danish cohort study on 313 children found that the number of respiratory infections in the first years of life, independently from the causative viral agents, was associated with asthma at the age of 7 years.^29^ As described earlier, hospitalization in early childhood because of viral wheezing episodes increased the risk of asthma in adulthood compared to controls in this cohort, and rhinovirus increased the risk more than other viruses.^17^

The prevalence of asthma in our cohort was much higher (53%) than that found in other prospective postbronchiolitis follow‐ups until adulthood conducted in Finland and Sweden, which reported asthma in 18% and 39%, respectively, of cohort subjects aged 18–29 years.^7^, ^8^, ^9^ There are no other, similar published prospective, longitudinal studies on patients hospitalized for viral wheezing episodes and followed up until adulthood. An increasing trend of asthma prevalence by time was clearly seen in these Finnish and Swedish studies; new studies reported a higher asthma prevalence than old studies. Thus, the prevalence of asthma in our study (53%) is in line with the current trend, although higher than expected since the allergy and asthma epidemics have decreased also in Nordic countries.^30^ In nonselected birth cohorts, the prevalence of asthma varied from 12% to 15% in longitudinal studies on adolescents and young adults aged 11–24 years.^31^ The prevalence of asthma among Finnish adults was 10.9% in a population‐based survey based on data collected using postal questionnaires.^30^ The higher prevalence of asthma among this cohort population might be due to participation bias—individuals with asthma‐related symptoms more likely to volunteer for follow‐up visits than those without symptoms.

Our cohort study had some limitations. The most important limitation was the small number of participants, which resulted in underpowered analyses and a risk of type‐2 errors. On the other hand, all the factors, such as previous asthma diagnoses at different ages through childhood, that showed statistically significant associations in the multivariate analyses in this small material, confirmed a real association with high asthma risk after early life viral wheezing episodes. Moreover, this cohort has been followed for more than 18 years, and therefore, dropouts are understandable. In terms of the strengths of the study, the cohort offers unique longitudinal data on the associations of viral wheezing episodes requiring hospitalization, and subsequent early childhood wheezing and asthma at preschool age, with asthma outcomes in adulthood.

In conclusion, this cohort study confirmed the results of previous studies that an asthma diagnosis at any age during childhood and an increased blood eosinophil count in early childhood are independent predictive factors for asthma in early adulthood.

## CONFLICTS OF INTEREST

The authors declare no conflicts of interest.

## AUTHOR CONTRIBUTIONS

*Design and conceptualization*: Matti Korppi, Katri Backman. *Formal analysis*: Paula Heikkilä. *Interpretation of the data*: Paula Heikkilä, Matti Korppi, Marja Ruotsalainen, and Katri Backman. Funding acquisition: Matti Korppi and Marja Ruotsalainen. *Writing—review and editing*: Matti Korppi, Marja Ruotsalainen, and Katri Backman. *Writing—original draft*: Paula Heikkilä. All authors have read and approved the final version of the manuscript. Paula Heikkilä had full access to all of the data in this study and takes complete responsibility for the integrity of the data and the accuracy of the data analysis.

## Supporting information

Supplementary information.

## Data Availability

The authors confirm that the data supporting the findings of this study can be obtained on request from the authors.
