# Supplementary material for: Viral wheezing in early childhood as a risk factor for asthma in young adulthood: A prospective long‐term cohort study
Source: Health Sci Rep. 2022 Mar 7;5(2):e538. doi: 10.1002/hsr2.538 (PMC8900980; doi:10.1002/hsr2.538)

**Supplementary data**

**Figure S1.** Asthma status during the 18-year follow-up of the original cohort (*N* = 100) of children hospitalized for viral wheezing episodes aged < 24 months.


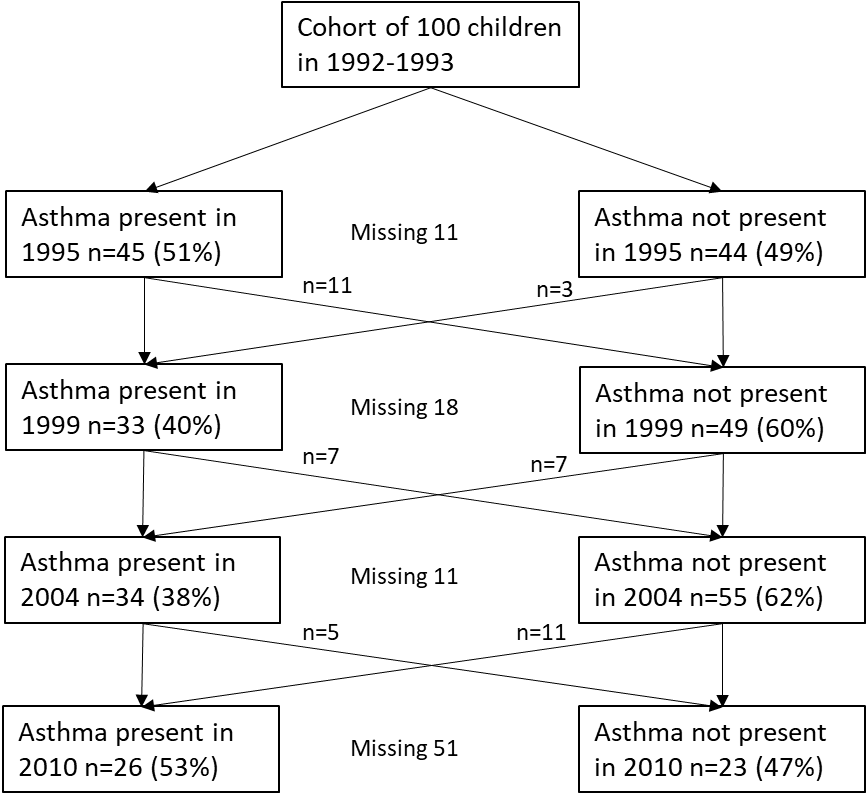

Supplement: Supplementary file 1 — Supplementary information. [file HSR2-5-e538-s001.docx]
